# A Novel Fibronectin Binding Motif in MSCRAMMs Targets F3 Modules

**DOI:** 10.1371/journal.pone.0005412

**Published:** 2009-04-30

**Authors:** Sabitha Prabhakaran, Xiaowen Liang, Jonathan T. Skare, Jennifer R. Potts, Magnus Höök

**Affiliations:** 1 Institute of Biosciences and Technology, Texas A&M Health Science Center, College Station, Texas, United States of America; 2 University of Texas Health Science Center Houston, Houston, Texas, United States of America; 3 Department of Microbial and Molecular Pathogenesis, Texas A&M Health Science Center, College Station, Texas, United States of America; 4 Department of Biology and Chemistry, University of York, York, United Kingdom; University of California Merced, United States of America

## Abstract

**Background:**

BBK32 is a surface expressed lipoprotein and fibronectin (Fn)-binding microbial surface component recognizing adhesive matrix molecule (MSCRAMM) of *Borrelia burgdorferi*, the causative agent of Lyme disease. Previous studies from our group showed that BBK32 is a virulence factor in experimental Lyme disease and located the Fn-binding region to residues 21–205 of the lipoprotein.

**Methodology/Principal Findings:**

Studies aimed at identifying interacting sites between BBK32 and Fn revealed an interaction between the MSCRAMM and the Fn F3 modules. Further analysis of this interaction showed that BBK32 can cause the aggregation of human plasma Fn in a similar concentration-dependent manner to that of anastellin, the superfibronectin (sFn) inducing agent. The resulting Fn aggregates are conformationally distinct from plasma Fn as indicated by a change in available thermolysin cleavage sites. Recombinant BBK32 and anastellin affect the structure of Fn matrices formed by cultured fibroblasts and inhibit endothelial cell proliferation similarly. Within BBK32, we have located the sFn-forming activity to a region between residues 160 and 175 which contains two sequence motifs that are also found in anastellin. Synthetic peptides mimicking these motifs induce Fn aggregation, whereas a peptide with a scrambled sequence motif was inactive, suggesting that these motifs represent the sFn-inducing sequence.

**Conclusions/Significance:**

We conclude that BBK32 induces the formation of Fn aggregates that are indistinguishable from those formed by anastellin. The results of this study provide evidence for how bacteria can target host proteins to manipulate host cell activities.

## Introduction

Different microbial pathogens use diverse sets of virulence factors to establish infections. In fact, the panels of virulence factors employed are often organism and host species specific and sometimes also disease specific. Despite this variation, bacterial infectious disease processes appear to involve some common steps. The microbial pathogen usually has to adhere to a tissue(s) in the host in order to establish a colony, and to survive, the microbe has to evade the host's defense systems.

In this context of diversity it is remarkable that so many pathogenic organisms appear to bind and adhere to fibronectin (Fn) substrates. Fn is an abundant animal glycoprotein found in a soluble form in most body fluids and deposited as part of the insoluble extracellular matrix of most tissues. The protein is composed of two 220 KDa polypeptides held together by disulfide bonds at the C-terminal end. Each polypeptide is a mosaic protein composed of three types of repeating modules: 12 type I modules (F1), 2 type II modules (F2), 15–17 (dependent on alternative splicing) type III modules (F3), and an alternatively spliced variable sequence that is not homologous to other parts of Fn. F1 and F2 modules are stabilized by disulfide bonds, whereas F3 modules lack disulfide bonds and can reversibly partially unfold [1], [2].

Soluble Fn adopts a somewhat compact form that is stabilized by intramolecular ionic interactions between specific modules. These interactions occur primarily between the ^1^F1–^5^F1, the ^2^F3–^3^F3, and ^12^F3–^14^F3 segments [3]. In the extracellular matrix, Fn takes on a more extended form and the protein is likely engaged in multiple intra- and intermolecular interactions (for review see [4]). The structural organizations of Fn matrices have not been elucidated in detail; however, it is likely that the incorporation of soluble Fn into a matrix involves a complex but orderly breaking of intramolecular bonds in the compact soluble Fn and the formation of new intra- and intermolecular interactions. This process is facilitated by integrins and other cellular components [5], [6]. Recently Vakonakis, *et al.*, suggested that cell-generated tension on Fn, disrupts interactions between ^1^F3 and ^2^F3, creating a conformation of ^1–2^F3 that binds to the N-terminal domain and initiates fibrillogenesis [7].

Fn play key roles in basic physiological processes such as cell proliferation, migration, differentiation, and survival by interacting with a variety of extracellular macromolecules and cellular receptors, primarily of the integrin family [8]. Integrins constitute a family of dimeric cell surface receptors that recognize specific extracellular matrix and cell bound ligands. Integrins α_5_β_1_, α_4_β_1_, and α_v_β_3_ are the primary Fn receptors.

Fn may occur in different aggregated forms. “Superfibronectin” (sFn) is a form of Fn aggregates that resembles Fn matrices, but with distinct biological activities [9]. sFn is formed by mixing plasma Fn with anastellin, a recombinant form of the C-terminal two-thirds of the ^1^F3 Fn module. F3 modules are β-sandwiches composed of a four-stranded β-sheet and a triple-stranded β-sheet. In anastellin, strands A and B are removed, exposing the E and C strands, which is perhaps responsible for anastellin's self-association and interaction with other Fn modules [10]. These interactions lead to the formation of sFn. Ohashi and Erickson suggested a model of sFn formation whereby anastellin binds and partially unfolds F3 modules and prevents refolding, thus exposing hydrophobic surfaces and β-sheet edges. The exposed elements bind to similar exposed elements on other Fn modules, leading to a specific aggregation [11]. sFn dramatically enhances cell adhesion compared to plasma Fn when coated on a cell culture dish [9], and it has been demonstrated that anastellin can inhibit angiogenesis. This effect is believed to represent the basis for anastellin's observed anti-tumor activity in mouse models of human cancers [12], [13]. McKeown-Longo, *et al*. showed that anastellin added to cultured cells becomes incorporated into the Fn matrix deposited by the cells and induces a conformational rearrangement in the Fn matrix that can be monitored by a specific monoclonal antibody. Anastellin also effectively blocks serum-dependent proliferation of cultured endothelial cells. This effect is not due to induced apoptosis, but is caused by blocking the serum-dependent activation of ERK [14].

More than 100 different Fn-binding microbial proteins have been reported so far, although most of the Fn-microbial protein interactions have not been characterized in detail. Previously, we showed that the Fn-binding MSCRAMMs, FnBPA and FnBPB from *Staphylococcus aureus* and Sfb1 from *Streptococcus pyogenes*, contain a common motif that allows these proteins to bind to the N-terminal domain (NTD) of Fn by a unique binding mechanism that we called the tandem β-zipper. When these MSCRAMMs target soluble Fn, the compact conformation of the host protein undergoes a change resulting in a more open structure, where binding sites for the α_5_β_1_ integrin are exposed (Liang, *et al.*, in preparation). Thus, staphylococci or streptococci recruiting Fn to their surfaces via these MSCRAMMs are coated with activated Fn that can lead to α_5_β_1_ integrin-dependent host cell invasion.

Fn-binding MSCRAMMs can also bind to other domains in Fn. A motif targeting the gelatin binding domain (GBD) in Fn has been identified in Sfb1. ShdA of *Salmonella enterica* serotype Typhimurium appears to target the F3 module, ^13^F3 (which makes up part of the heparin-2-binding domain of Fn) [15]. The binding motif in ShdA has not been clearly defined. Furthermore, the biological consequences of these interactions are still unclear.

We have unsuccessfully searched many of the identified Fn-binding MSCRAMMs for the presence of the motifs identified from FnBPA, FnBPB, and Sfb1 that target the Fn NTD or GBD. An exception is BBK32 from *Borrelia burgdorferi*. BBK32, a 47 kDa lipoprotein, was originally identified as a Fn-binding MSCRAMM by probing lysates of spirochete with Fn in Western ligand blots [16]. It was localized to the surface of the spirochete and the attachment of *B. burgdorferi* to Fn substrates was inhibited by the addition of soluble recombinant BBK32 protein suggesting that BBK32 is a Fn-binding adhesin on *B. burgdorferi* sensu stricto [16]. Orthologous genes are found in the closely related species, *B. garinii* and *B. afzelii* [17]. Expression of BBK32 at the site of experimental infection in mice increases until day 7 and then declines, but *bbk32* gene expression can be detected in the skin, heart, spleen, joints, and bladder at least 30 days post challenge, indicating that the lipoprotein is expressed by the spirochete as it disseminates to different tissues in the host [18].

The N-terminus of BBK32 contains a signal peptide followed by a “lipobox” and an extended intrinsically disordered segment (residues 21–205) that contains the Fn-binding sites [19]. This segment contains a sequence motif that resembles the motifs in FnbpA. In fact, BBK32_(147–205)_ binds to the N-terminal type I modules found in Fn by the tandem β-zipper mechanism [20]. In addition, we identified a motif resembling the GBD-binding sequence in Sfb1 [20].

In further analysis of the binding specificity, we found that the MSCRAMM not only binds to the NTD F1 modules [19], [20] and the GBD (manuscript in preparation), but also the ^1–2^F3, and ^3^F3 modules. This observation prompted us to examine if BBK32, like anastellin can induce conformational changes in soluble Fn that can lead to aggregation of the glycoprotein. Here we report that the BBK32-F3 interaction induces an ordered aggregation of soluble Fn that exposes thermolysin cleavage sites that are cryptic in soluble Fn. Recombinant BBK32 has specific biological activities in that it can affect the structure of Fn matrices formed by cultured fibroblasts and effectively inhibit endothelial cell proliferation. Furthermore, we have identified two specific amino acid sequence motifs found in BBK32 that can induce Fn aggregation. Thus, we conclude that a specific motif in BBK32 can target F3 modules in Fn and induce the formation of ordered Fn aggregates.

## Results

### BBK32 binds to ^1–3^F3 of Fn

**Figure 1:**
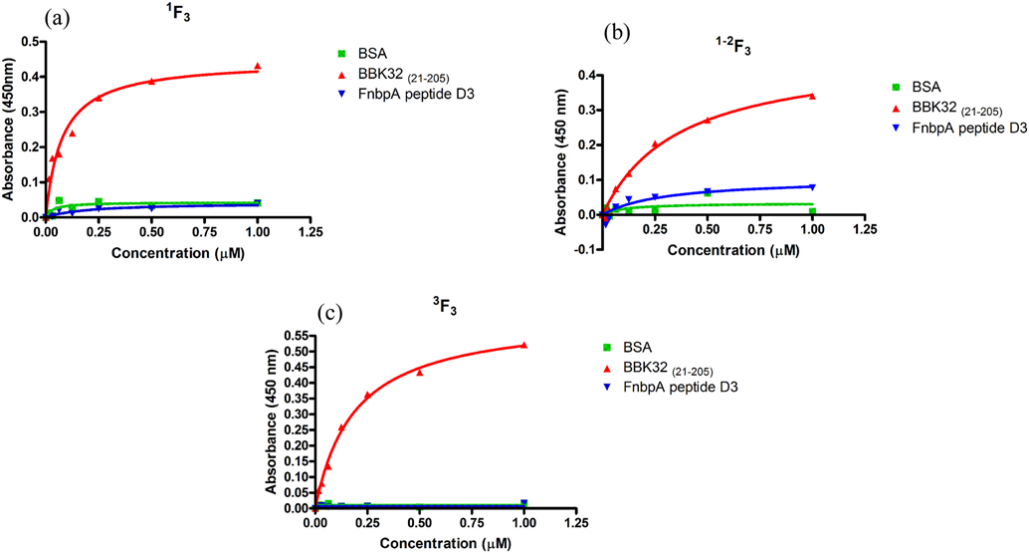
BBK32 _(21–205)_ binds F3 modules. Binding to (a) ^1^F3 (b) ^1–2^F3 and (c) ^3^F3 was assessed using ELISA-type assays. F3 modules were immobilized in microtiter wells followed by blocking with ovalbumin. BBK32 _(21–205)_ or FnbpA peptide D3 was added to wells in increasing concentrations. Anti-BBK32 antibody followed by HRP conjugated anti-rabbit antibody for BBK32 _(21–205)_, and anti-FnbpA followed by HRP conjugated anti-rabbit antibody were used to demonstrate binding.

We previously reported that the Fn-binding activity of BBK32 is located to an intrinsically disordered segment of the protein corresponding to residues 21–205 [19] and that a motif corresponding to residues 147–205 binds to the ^1^F1–^5^F1 N-terminal segment of Fn by the tandem β-zipper mechanism [19], [20]. Experiments designed to further define the MSCRAMM binding sites in Fn by probing a thermolysin digest of Fn with rBBK32 _(21–205)_ in Western ligand blots to determine the protein-protein interaction, indicated that the MSCRAMM binds more strongly to the 56 kDa fragment than to the 43 kDa gelatin binding fragment (data not shown). Since the only difference between these two Fn fragments is the inclusion of ^1^F3 in the 56 kDa fragment, this result suggests that BBK32 _(21–205)_ may interact with the ^1^F_3_ module. We, therefore, generated recombinant forms of ^1^F3, ^1–2^F3, and ^3^F3 and examined the binding of BBK32 to these Fn modules in ELISA-type binding assays. BBK32 bound all the recombinant F3 modules in a concentration dependent manner. The binding to the F3 module was specific for BBK32 as the D3 fragment of *S. aureus* FnbpA, which binds ^1^F1–^5^F1, did not to bind the F3 modules (Fig. 1). We calculated half-maximal binding concentrations of 76.9 nM, 353 nM, and 188.1 nM for the binding of rBBK32 _(21–205)_ to the ^1^F3, ^1–2^F3, and ^3^F3 respectively, indicating that the MSCRAMM has a high affinity for the immobilized F3 modules.

### BBK32 aggregates plasma Fn in a concentration dependent manner

**Figure 2:**
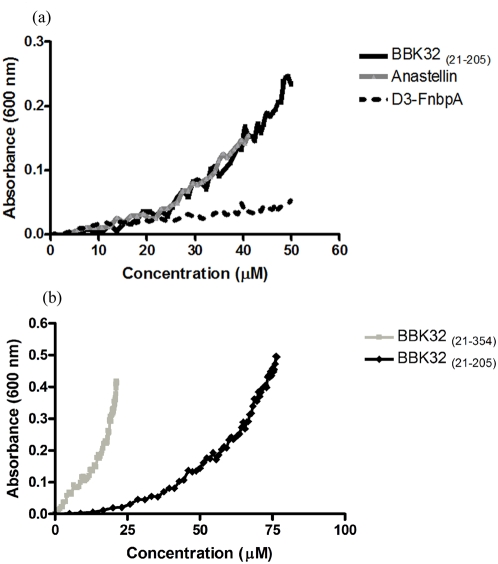
BBK32 _(21–205)_ and BBK32 _(21–354)_ aggregate plasma Fn. (a) BBK32 _(21–205)_, FnbpA peptide D3, and anastellin (b) or BBK32 _(21–354)_ and BBK32 _(21–205)_ were titrated into 1 µM purified plasma Fn and the optical density at 600 nm was measured.

The interaction of BBK32 with ^1^F3, ^1–2^F3, and ^3^F3 is particularly interesting since anastellin, an inducer of sFn, also binds to these modules [21]. This similarity raises the possibility that BBK32 could induce a form of Fn aggregation. To test this possibility we incubated 1 µM of purified plasma Fn with increasing concentrations of rBBK32 _(21–205)_, anastellin, or the FnbpA D3 fragment. BBK32 and anastellin, but not the D3 protein induced Fn aggregation that could be followed by the increase in absorbance at 600 nm (Fig. 2a). Fn aggregation was dependent on the concentration of the inducer, and anastellin and rBBK32 _(21–205)_ showed very similar activity with measurable aggregation initiated at 20 µM of the inducer. Aggregation of Fn was also observed with full length BBK32, which includes the C-terminal globular domain (residues 21–354) (Fig. 2b). rBBK32 _(21–354)_ was more active and induced aggregation of Fn at lower concentrations than rBBK32 _(21–205)_. However, since the longer form of BBK32 is more susceptible to degradation, we used rBBK32 _(21–205)_ to further characterize the BBK32-dependent Fn aggregation.

**Figure 3:**
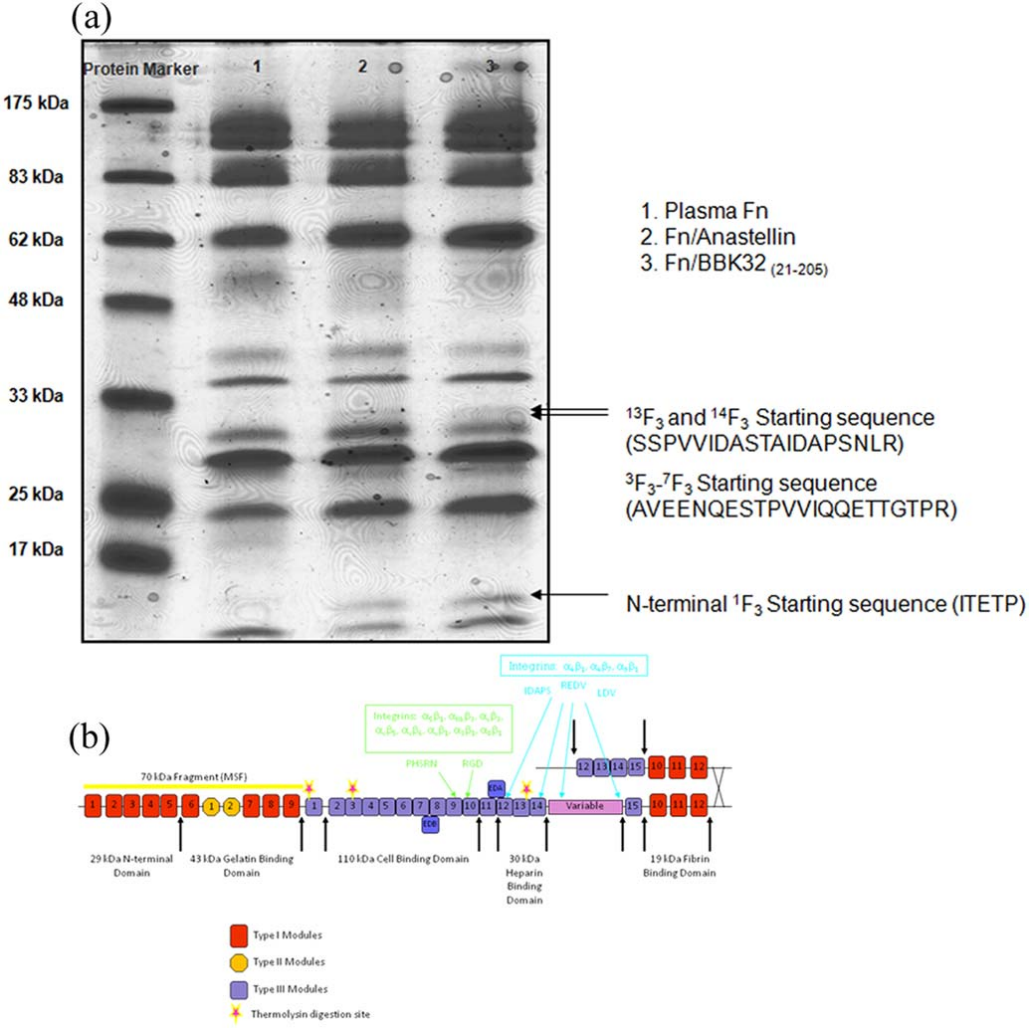
BBK32 _(21–205)_ induces a conformational change in plasma Fn. (a) Purified plasma Fn and Fn aggregates induced by anastellin or BBK32 _(21–205)_ were digested with thermolysin. The digested products were fractionated by SDS-PAGE and peptides that were only found in BBK32 _(21–205)_ /Fn (lane 3) or anastellin/Fn (lane 2) digests and not present among the peptides obtained from purified plasma Fn (lane 1) were excised and identified by N-terminal sequencing. The sequences obtained, SSPVVID, AVEENQE, and ITETIP, were located to the end of ^13^F3, middle of ^3^F3, and the start of ^1^F3, respectively for both BBK32/Fn and anastellin/Fn. Pre-stained protein standards with indicated M_w_ are shown. (b) Schematic of Fn. Stars denote cryptic cleavage sites in Fn after aggregation due to both BBK32 and anastellin.

Initial experiments showed that addition of a detergent, Triton X-100, inhibited Fn aggregation by BBK32 (not shown) as was previously reported for anastellin induced Fn aggregation [11]. When plasma Fn is induced to aggregate by anastellin the protein apparently undergoes conformational changes. These changes involve exposure of cryptic thermolysin cleavage sites which result in unique fragments generated by enzymatic digestion [11]. To determine if BBK32 elicits a similar effect, we digested purified plasma Fn and Fn aggregates induced by anastellin or rBBK32 _(21–205)_ with thermolysin and fractionated the resulting peptides by SDS-PAGE. As seen in figure 3, digestion of Fn aggregates induced by anastellin or BBK32 resulted in very similar fragment profiles. Furthermore, some of the generated peptides are uniquely found in the rBBK32_(21–205)_/Fn or anastellin/Fn digests and are not present among the peptides obtained from digested purified plasma Fn. The bands indicated by arrows in the SDS-PAGE (and equivalent sites from the plasma Fn lanes) were excised and subjected to N-terminal sequencing. The sequences obtained, SSPVVID, AVEENQE, and ITETIP, were located at the C-terminus of ^13^F3, the middle of ^3^F3, and near the N-terminus of ^1^F3 respectively (Fig. 3b). N-terminal sequencing of the material recovered from the corresponding space from the plasma Fn only digest did not detect these sequences. Taken together, these results suggest that aggregation of plasma Fn by BBK32 or anastellin involves similar types of conformational changes in the glycoprotein and that these conformational changes are not limited to the ^1–3^F3 region but extend towards the C-terminus of the protein.

### BBK32 affects the structural organization of Fn matrix assembly

The addition of Fn-binding MSCRAMMs or anastellin to fibroblast cultures results in the incorporation of the exogenous proteins into the extracellular matrix formed by the cultured cells. BBK32, anastellin, and FnbpA peptide D3 co-localize with Fn in the extracellular matrix of normal human dermal fibroblasts (data not shown).

**Figure 4:**
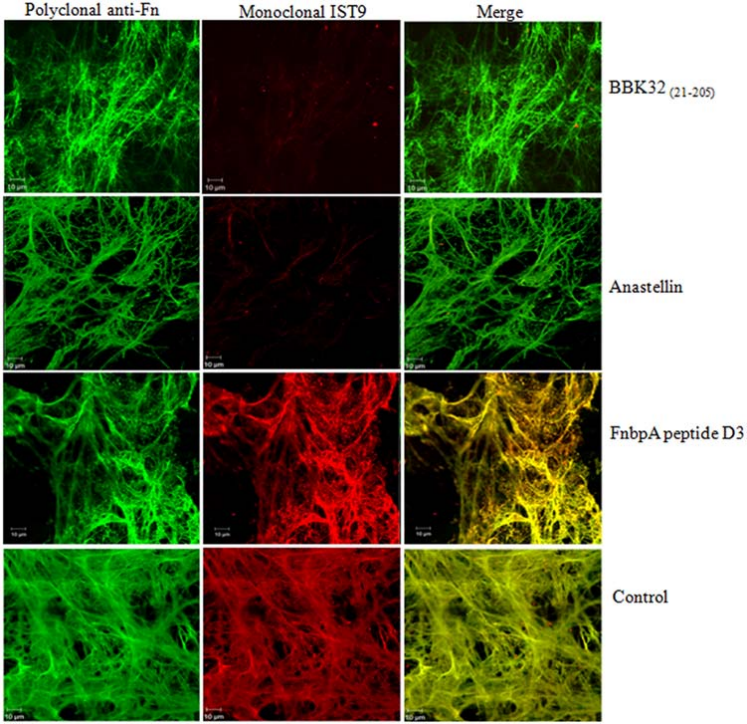
BBK32 _(21–205)_ effects on Fn matrix assembled by NHDFs. Cells were incubated for 48 hours and then incubated with 5 µM BBK32 _(21–205)_, 5 µM anastellin, or 5 µM FnbpA peptide D3. Treated cells were incubated for 20 hours and probed with Alexa Fluor 488-anti-Fn and monoclonal antibody IST 9. Images were taken using LSM 510 Confocal Microscope, objective 63X/1.4 oil. Bar = 10 µm.

The interaction of anastellin and Fn results in conformational changes in cellular Fn that can be monitored using a monoclonal antibody specific to the alternatively spliced extra domain A (EDA). EDA is only expressed in cellular Fn and is upregulated during embryogenesis, wound healing, and tumor progression. Monoclonal antibody IST 9, which targets residues ^39^PEDGIHELFP^48^ found in the C-C' loop of EDA [22], [23], does not recognize cellular Fn in the presence of anastellin [24]. Binding of anastellin to Fn appears to induce a conformational change in EDA resulting in the loss of the IST 9 epitope. As seen in figure 4, the addition of anastellin (5 µM) to cultured NHDFs results in markedly reduced immunostaining with IST 9. Addition of rBBK32_(21–205)_ (5 µM) also results in a reduction of immunostaining with IST 9 (Fig. 4). The D3 fragment of FnbpA was used as a control. IST 9 staining of D3 treated cells was no different from the staining observed for the no treatment control (Fig. 4). Polyclonal anti-Fn and monoclonal IST 1 (not shown), which recognizes an epitope in ^12^F_3_, were used to demonstrate the presence of Fn in BBK32 or anastellin treated matrices. The marked reduction in immunostaining with IST 9 indicates that BBK32 may alter the conformation of cellular Fn in a manner similar to that seen with anastellin.

### BBK32 inhibits the proliferation of cultured endothelial cells

**Figure 5:**
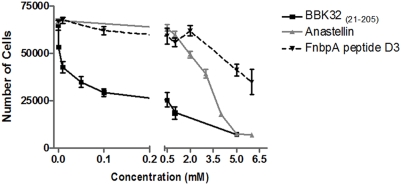
BBK32 _(21–205)_ inhibits the proliferation of HUVECs. Cells were seeded onto tissue culture plates in full media and allowed to adhere for two hours. After two hours, BBK32, anastellin, and FnbpA peptide D3 were added in increasing concentrations (in triplicate) to the wells. Cells were then incubated for 72 hours at 37°C, 5% CO_2_. After 72 hours, cells were washed, trypsinized, and counted (three counts/well, three wells/dose) using a hemacytometer.

The ability of anastellin to inhibit endothelial cell proliferation is one of its most prominent activities [13], [14], [25]. Since BBK32 behaves like anastellin in its interaction with Fn, we examined the ability of BBK32 to affect proliferation of human umbilical vein endothelial cells (Fig. 5). As before, we used anastellin and the D3 fragment of FnbpA as controls. The results show that BBK32 inhibited the proliferation of endothelial cells in a concentration dependent manner as observed for anastellin. Surprisingly, BBK32 was reproducibly more potent at inhibiting endothelial cell proliferation than anastellin. The decrease in proliferation was observed at 24 and 48 hours (data not shown), but was more pronounced following 72 hours of static culture. The FnbpA peptide D3 only marginally affected the proliferation of cells.

The inhibition of endothelial cell proliferation was apparently not due to apoptosis since Annexin V assays were negative (not shown). Ambesi *et al.* demonstrated that anastellin inhibits endothelial cell proliferation by causing G1 arrest [14]. Since BBK32 does not induce apoptosis and there is no evidence of cell death, it is tempting to speculate that the BBK32-dependent inhibition of endothelial cell proliferation may also be due to cell cycle arrest.

### Locating the sFn-inducing site in BBK32

Since the Fn-binding region of BBK32 corresponding to residues 21–205 has a disordered structure it is reasonable to assume that a linear sequence is responsible for the sFn inducing activity. To locate this hypothetical motif, we first examined the activity of shorter recombinant fragments. Using ELISA-type solid phase binding assays, we identified a F3-binding site to a region between BBK32 residues 146 and 205 (data not shown). We then synthesized and examined the activity of a panel of peptides covering the segment between residues 146 and 202 (Table 1).

**Table 1:** Panel of synthetic BBK32 peptides used in experiments.

Construct	Residues	Aggregate Fn	Inhibit Proliferation
rBBK32 _(145–205)_	EEPIESNEIDLTIDSDLRPKSSLQGIAGSNSISYTDEIEEEDYDQYYLDEYDEEDEEEDYD	Yes	Yes
sBBK32 _(146–166)_	EPIESNEIDLTIDSDLRPKSS	No	Yes
sBBK32 _(153–175)_	IDLTIDSDLRPKSSLQGIAGSNS	Yes	Yes
sBBK32 _(175–202)_	SISYTDEIEEEDYDQYYLDEYDEEDEEE	No	No
sBBK32 _(160–186)_	DLRPKSSLQGIAGSNSISYTDEIEEED	Yes	Yes
sBBK32 _(160–186)scramble_	GSELYESDISPENKIGQSDISRDTELA	No	No
sBBK32 _(153–166)_	IDLTIDSDLRPKSS	No	No
sBBK32 _(160–175)_	DLRPKSSLQGIAGSNS	Yes	Yes
sBBK32 _(160–167)_	DLRPKSSL	Yes	Not Tested
sBBK32 _(168–175)_	QGIAGSNS	Yes	Not Tested

**Figure 6:**
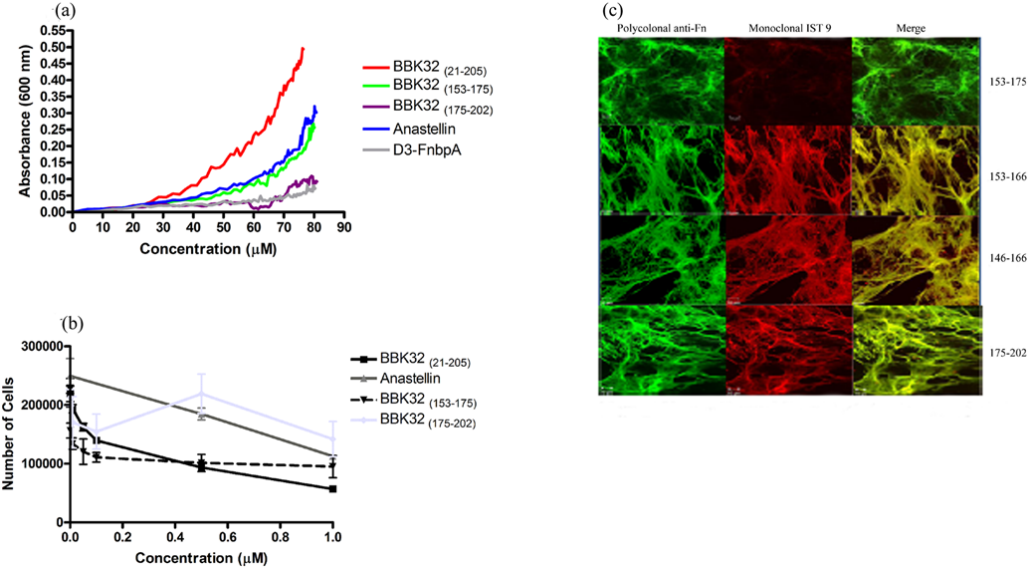
BBK32 _(153–175)_ aggregates plasma Fn. (a) BBK32 _(21–205)_, BBK32 _(146–166)_, BBK32 _(153–175)_, and BBK32 _(175–202)_ were titrated into 1 µM purified plasma Fn and the optical density at 600 nm was measured. (b) BBK32 _(153–175)_ is the minimal amino acid sequence required to affect Fn matrices assembled by NHDFs. Cells were allowed to grow for 48 hours and then were incubated with 5 µM BBK32 _(153–166)_, BBK32 _(146–166)_, BBK32 _(153–166)_, and BBK32 _(175–202)_. Treated cells were incubated for 20 hours and probed with Alexa Fluor 488-anti-Fn and monoclonal antibody IST 9. Images were taken using LSM 510 Confocal Microscope, objective 63X/1.4 oil. Bar = 10 µm. Staining with monoclonal antibody IST 9 indicates that only the BBK32 peptide containing residues 153–175 changes the conformation of Fn matrices. (c) BBK32 _(153–175)_ is the minimal amino acid sequence required to inhibit HUVEC proliferation. Cells were seeded onto tissue culture plates in full media and allowed to adhere for two hours. After two hours, BBK32 _(21–205)_, BBK32 _(153–175)_, BBK32 _(175–202)_, and anastellin were added in increasing concentrations (in triplicate) to the wells. Cells were then incubated for 72 hours at 37°C, 5% CO_2_. After 72 hours, cells were washed, trypsinized, and counted (three counts/well, three wells/dose) using a hemacytometer.

sBBK32_(153–175)_ aggregated plasma Fn in a concentration-dependent manner and was essentially as active as anastellin, whereas the other two peptides (sBBK32_(146–166)_ and sBBK32 _(175–202)_) had minimal activity (Fig. 6a). The addition of 5 µM BBK32 _(153–175)_ resulted in a reduction of immunostaining with IST 9. On the other hand, BBK32 peptides 146–166, 153–166, and 175–202 did not affect monoclonal antibody IST 9 recognition of EDA (Fig. 6b). Addition of the BBK32 peptide 153–175 resulted in inhibition of endothelial cell proliferation (Fig. 6c). In fact, this peptide was consistently more active than anastellin or BBK32 _(21–205)_. Peptide 175–202 had a minimal effect on HUVEC proliferation. Taken together our data suggest that the sFn-inducing activity in BBK32 is located to a site between residues 160–175.

### A conserved sequence motif in BBK32 and anastellin induces ordered Fn aggregation

**Figure 7:**
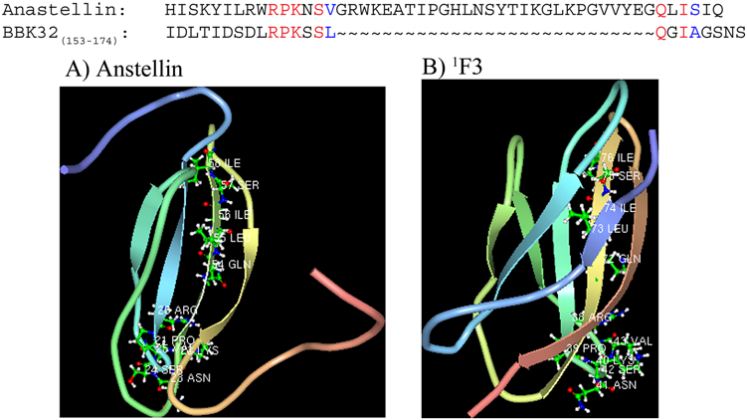
Amino acid sequence alignment of BBK32_(153–175)_ and anastellin demonstrates weak homology. (A) NMR structure of anastellin demonstrating homologous amino acids and (B) NMR structure of ^1^F3.

**Figure 8:**
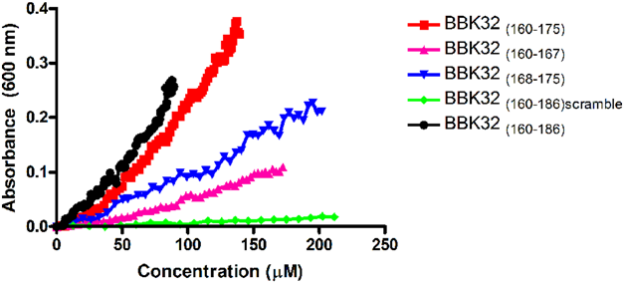
BBK32 _(160–175)_ aggregates plasma Fn. BBK32 _(160–175)_, BBK32 _(160–167)_, BBK32 _(168–175)_, BBK32 _(160–186)_, and BBK32 _(160–186)scramble_ were titrated into 1 µM purified plasma Fn and the optical density at 600 nm was measured.

Alignment of the 160–175 segment of BBK32 and anastellin revealed two distinct regions of weak sequence similarity (Fig. 7). In order to further delineate the specific BBK32 motif capable of aggregating Fn, we examined three additional synthetic peptides (residues 160–175, 160–168, and 168–175) that correspond to the intact or split motif conserved between anastellin and BBK32. Synthetic BBK32 peptides 160–186, 160–175, 160–167, and 168–175 all caused Fn to aggregate as measured by optical density at 600 nm. Peptide 160–175 was the most effective aggregation inducer (Fig. 8), but both sBBK32 _(160–167)_ and sBBK32 _(168–175)_ induced Fn aggregation in our assay. In addition, a scramble peptide including the entire active motif (residues 160–186) is unable to aggregate Fn. NMR data indicate that in anastellin, the two active motifs are separated by 28 amino acids, but when the protein is folded, the two sequences are in close proximity to each other (Fig. 7). One of the active motifs in anastellin is found on a β-strand that is not exposed in the ^1^F3 module (Fig. 7), whereas the other motif is found in a short loop.

## Discussion

The *B. burgdorferi* lipoprotein BBK32 was previously shown to bind to the N-terminal ^1^F1–^5^F1 segment of Fn through a tandem β-zipper mechanism [19], [20]. We now demonstrate that a recombinant form of BBK32 _(21–205)_ also binds to Fn F3 modules and can induce an ordered Fn aggregation that resembles the formation of sFn induced by anastellin. This aggregation probably is a consequence of conformational changes induced in Fn upon BBK32 or anastellin binding. Aggregates of plasma Fn induced by either BBK32 or anastellin expose similar thermolysin cleavage sites. Both proteins affect the structure of cellular Fn in deposited matrices by cultured fibroblasts as monitored by the disappearance of the epitope for the monoclonal antibody IST9. Furthermore, both BBK32 and anastellin can inhibit the proliferation of cultured endothelial cells. Thus, BBK32 and anastellin appear to modulate the conformation of Fn in similar ways and have similar biological activities. The ability to induce ordered Fn aggregation is not a property seen for all Fn-binding MSCRAMMs. A segment of the staphylococcal Fn-binding MSCRAMM FnbpA did not show these effects. The FnbpA D3 segment contains a well defined ^4^F1–^5^F1 binding motif, but does not bind to F3 modules nor does it induce Fn aggregation.

The Fn-binding activity of BBK32 was previously located to an intrinsically disordered segment of the lipoprotein corresponding to residues 21–205. The sequence 147–205, found within the segment, binds the Fn NTD and resembles the NTD-binding motifs identified in FnbpA of *S. aureus* and Sfb1 of *S. pyogenes*. BBK32 also includes a putative gelatin binding domain (GBD)-binding motif corresponding to residues 120–147 [20] that is similar to a motif in Sfb1 that has GBD-binding activity [26], [27], [28]. Now, we have identified a F3 binding motif in the BBK32 Fn-binding segment. This F3-binding motif is located within residues 147–205. It is interesting to note that the NTD-binding motif in BBK32 also is located to residues 147–205. Residues 165–182 contain the ^2^F1^3^F1 binding motif and residues 146–162 contain the ^4^F1^5^F1 binding motif [20]. Soluble Fn is stabilized by intramolecular ionic interactions between specific modules. These interactions occur among the ^1^F1–^5^F1, the ^2^F3–^3^F3, and the ^12^F3–^14^F3 segments [3]. Binding to the NTD might cause a conformational change in the F3 modules that could then be stabilized by a BBK32-F3 module interaction leading to Fn aggregation.

The F3-binding motif from BBK32 is also present in anastellin. The residues in BBK32 that appear to comprise the sFn-inducing motif have weak sequence homology to a sequence found in anastellin (Fig. 8). Recombinant anastellin and BBK32 are structurally different. Anastellin represents the C-terminal two-thirds of the Fn ^1^F3 module, and has a defined structure with two β-sheets. When both proteins are aligned the motif appears to be divided, however, when anastellin is properly folded, the residues are in close proximity and are available for Fn interactions (Fig. 8). The active sFn-inducing motif in anastellin has not yet been elucidated, however, residues QLISI (weak homology to the QGIAG sequence in BBK32 _(168–175)_), are only exposed in anastellin and not in the complete ^1^F3 module. The RPKNSV sequence identified in anastellin is in a short loop region of the protein where it could have a restricted flexibility in the intact F3 module. In anastellin, the ^1^F3 module is truncated. This truncation might lead to a partial unfolding which might release the structural constraints of the fold. BLAST [29] searches using the intact or divided F3-binding motifs as probes reveal a number of microbial and mammalian proteins containing related motifs (data not shown). Some of the identified proteins are known to bind Fn. These include the microbial proteins Efb from *Staphylococcus aureus* [30] and Sfb1 *Streptococcus pyogenes* [31] and a segment in human type I collagen which induces Fn fibrillogenesis [32].

We have shown that full length BBK32 (residues 21–354) is active and can induce sFn formation. It should be pointed out that this is the first native protein shown to have sFn-inducing activity. Anastellin is an artificial, recombinant fragment that is probably not present *in vivo*. Although it is yet unknown whether intact BBK32 expressed on the surface of *B. burgdorferi* induces sFn, we can speculate about potential biological consequences of BBK32-induced-sFn formation. The ability of BBK32 to inhibit endothelial cell proliferation, which may be a function of sFn, may ultimately facilitate the hematogenous spread of the spirochetes that are initially locally deposited at the tick bite site. During this process the spirochetes have to cross the endothelial lining twice, once to enter the blood stream and once to exit. Elegant studies by Norman, *et al.*, demonstrated, using intravital microscopy, that a non-infectious strain of *B. burgdorferi* lacking BBK32, among other lipoproteins (VlsE, OspF, Erpl, and ErpK), had fewer transient interactions, dragging interactions, and stationary adhesions per minute with the endothelium in a live murine infection model. When the non-infectious strain of *B. burgdorferi* was complemented with BBK32, the numbers of interactions per minute were restored to the same levels of the infectious strain [33]. This indicates that BBK32 plays an important role in *B. burgdorferi*-endothelium interactions. The results of this study correlate well with earlier studies that demonstrated endothelial cell attachment and internalization of *B. burgdorferi in vitro* [34], [35], [36], [37], [38]. In addition, in a mouse model of Lyme disease, *B. burgdorferi* mutant strains containing *bbk32* gene deletions have an attenuated infectivity when assessed at twenty-one days post infection [39], demonstrating that BBK32 is a virulence factor in borrelioses.

It is also intriguing that BBK32 induces a conformational change in tissue Fn that can be monitored by the loss of the epitope recognized by the monoclonal antibody IST 9. This epitope has been located to the EDA module (specifically residues ^39^PEDGIHELFP^48^) that also harbors binding sites for the α_4_β_1_ and α_9_β_1_ integrins [40]. Thus, it is possible that binding of BBK32 to Fn may regulate Fn's interactions with these integrins, which in turn may benefit the spirochetes. The effects of BBK32 on Fn interactions with different integrins are currently being investigated in our laboratory.

## Materials and Methods

### Proteins

The anastellin construct was generously provided by Dr. Paula McKeown-Longo (Albany Medical College). A recombinant protein (amino acids: NAPQPSH….LISIQQ) with a C-terminal His-tag was produced in bacteria and purified [9], [24], [25]. Recombinant BBK32 _(21–205)_ was expressed and purified as described [19]. Recombinant (r) F_3_ modules (amino acids: ^1^F3: SGPVE….DFTTT, ^1–2^F3: SGPVE …. TSQTT, and ^3^F3: APDAP …. QETTG) were expressed with N-terminal His-tags and purified by standard Ni-NTA and ion-exchange chromatography. FnbpA peptide D3 (HGFN….LPKV), synthetic (s) BBK32 _(146–166)_, sBBK32 _(153–166)_, sBBK32 _(153–175)_, sBBK32 _(175–202)_, sBBK32 _(160–186)_, and sBBK32 _(160–186)scramble_ were synthesized and purified by HPLC using previously described methods [41]. sBBK32 peptides 160–175, 160–167, and 168–175 were synthesized and purified to at least 98% by BioMatik Corporation (Cambridge, ON, Canada). Molecular weights were determined by mass spectrometry (Tufts Core Facility, Boston, MA). Plasma Fn was affinity purified from human plasma (Gulf Coast Regional Blood Center Houston, TX) as described previously [42]. Protein concentrations were calculated using absorbance at 280 nm.

### ELISA-type binding assay

Binding of BBK32 to F3 modules was tested by coating 1 µg of recombinant ^1^F3, ^1–2^F3, and ^3^F3 modules in 50 µl of 50 mM Tris-HCl, 150 mM NaCl, pH 7.5 (TBS) per well in Immulon 4HBX microtiter plates (Dynatech Laboratories, Chantilly, VA) and incubating overnight at 4°C. The next day, the wells were washed with 200 µl TBS, 0.05% Tween 20, pH 7.4 (TBST) and blocked with 200 µl TBST containing 2% ovalbumin (Sigma, St. Louis, MO) for one hour at room temperature. The wells were then washed and rBBK32 _(21–205)_ or peptide D3 from FnbpA was added to the wells in increasing concentrations. Plates were incubated for one hour at room temperature. After washing with TBST, primary antibodies (rabbit IgG anti-BBK32 or rabbit IgG anti-FnbpA, produced by Rockland, Gilbertsville, PA) were added to each well at a concentration of 0.48 µg/ml. Plates were incubated for one hour at room temperature. Following washing with TBST, a 1∶3000 dilution of HRP-conjugated goat-anti-rabbit antibodies (BioRad, Hercules, CA) was added to each well. Plates were incubated for one hour at room temperature and then washed with TBST. Color was developed by adding SigmaFast^™^ OPD (Sigma) to each well and incubating at room temperature for 10 minutes. Absorbance was measured at 450 nm using a Thermo Max microplate reader (Molecular Devices, Sunnyvale, CA). Half-maximal binding was assessed using GraphPad Prism 4.

### Fn aggregation assay

rBBK32 _(21–354)_, rBBK32 _(21–205)_, sBBK32 _(146–166)_, sBBK32 _(153–175)_, sBBK32 _(175–202)_, sBBK32 _(160–167)_, sBBK32 _(160–175)_, sBBK32 _(168–175)_, sBBK32 _(160–186)_, sBBK32 _(160–186)scramble_, FnbpA peptide D3, anastellin, and purified plasma Fn were centrifuged at 14,000 rpm for 15 minutes and then filtered through 0.22 µm PES syringe filters (Nalgene, Rochester, NY). Following filtration, BBK32, FnbpA peptide D3, and anastellin were titrated into 1 µM purified plasma Fn in TBS. After the addition of each aliquot, the Fn/protein mixture was carefully mixed five times with a 200 µl pipette and optical density at 600 nm was measured using a BioPhotometer (Eppendorf, New York, NY) after 10 seconds.

### Proteolytic digestion of Fn aggregates

BBK32, anastellin, and plasma Fn were dialyzed into thermolysin digestion buffer (25 mM Tris, 0.5 mM EDTA, pH 8, 50 mM NaCl, and 2.5 mM CaCl_2_). 20 µM BBK32 or 20 µM anastellin was added to 1 µM plasma Fn. Solutions were mixed and aggregates were allowed to form for one hour at room temperature. After one hour incubation, 5 µg/ml thermolysin (Sigma) was added to the BBK32/Fn, the anastellin/Fn, and to plasma Fn. The solutions were incubated at room temperature with end-over-end mixing for 2 hours. Digestion was stopped after two hours with the addition of EDTA to a final concentration of 5 mM. Samples were then run on 4–20% gradient SDS-PAGE gels under non-reducing conditions. Protein fragments were transferred to PVDF membranes (Millipore, Billerica, MA) and stained with 0.25% Coomassie Brilliant blue-250 in 40% methanol until bands were visible. Excess stain was removed with 40% methanol and bands that were present in BBK32/Fn and anastellin/Fn, but not in plasma Fn lanes, were excised and analyzed by N-terminal sequencing using Edman degradation (Protein Sequencing Division, Tufts University Core Facility, Boston, MA).

### BBK32 effects on Fn matrix assembled by normal human dermal fibroblasts

4×10^4^ adult normal human dermal fibroblasts (NHDFs) (Cambrex, East Rutherford, NJ) were seeded onto glass coverslips in 24 well Costar tissue culture plates in fibroblast growth media-2 (FGM-2) (Cambrex). Cells were incubated for 48 hours and then rBBK32 _(21–205)_, sBBK32 _(146–166)_, sBBK32 _(153–175)_, sBBK32 _(175–202)_, anastellin, or FnbpA peptide D3 was added to a final concentration of 5 µM in FGM-2. Treated cells were incubated for 20 hours at 37°C, 5% CO_2_. After incubation, cells were washed with Dulbecco's PBS (DPBS) (Gibco, Invitrogen, Carlsbad, CA) and fixed with 2% paraformaldehyde for 30 minutes at room temperature. Following washing with DPBS, cells were blocked with 1% BSA for one hour at room temperature. Cells were then probed with Alexa Fluor 488-anti-Fn, and monoclonal antibodies IST-9 (cellular Fn) (abcam, Cambridge, UK) or combinations of antibodies at a dilution of 1∶3000 for one hour at room temperature. After incubation, cells were washed extensively with DPBS and coverslips were mounted using Gel/Mount (biømeda, Foster City, CA) onto glass slides and images were taken using a LSM 510 Confocal Microscope (Zeiss).

### Endothelial cell proliferation assay

1×10^4^ human umbilical vein endothelial cells (HUVECs) (Cambrex) were seeded into Costar 24 well tissue culture plates in endothelial growth media-2 (EGM-2) (Cambrex) and allowed to adhere for two hours. After two hours, rBBK32 _(21–354)_, rBBK32 _(21–205)_, sBBK32 _(146–166)_, sBBK32 _(153–175)_, sBBK32 _(175–202)_, anastellin, and FnbpA peptide D3 were added in a dose dependent manner to the wells. Cells were incubated for 72 hours at 37°C, 5% CO_2_. After 72 hours, wells were washed with PBS and cells were trypsinized with 0.5% trypsin/EDTA (Cambrex). Trypsinized cells were collected in microcentrifuge tubes and trypsin/EDTA was neutralized with EGM-2. Cells were counted from each well (three counts/well, three wells/dose) using a hemacytometer.
